# Aeroallergen sensitization patterns among patients with chronic rhinitis with or without concomitant asthma

**DOI:** 10.1016/j.bjorl.2023.101351

**Published:** 2023-10-27

**Authors:** Tássia Milenna Oliveira de Souza, Jamille Souza Fernandes, Cinthia Vila Nova Santana, Marcus Miranda Lessa, Álvaro A. Cruz

**Affiliations:** a Universidade Federal da Bahia, Salvador, BA, Brazil; b Universidade Federal do Oeste da Bahia, Barreiras, BA, Brazil; c Faculdade Bahiana de Medicina e Saúde Pública, Salvador, BA, Brazil

**Keywords:** Allergic sensitization, Chronic rhinitis, Asthma, Severe asthma, Indoor allergens

## Abstract

**Objectives:**

The aim of this study was to explore the differences in the pattern of allergen sensitization in CR individuals without or with asthma, according to asthma severity.

**Methods:**

A total of 1066 adults were evaluated. Asthma and chronic⁄allergic rhinits were identified by specialists, questionnaries and skin-prick test. The phenotypic characterization was avaliable from skin-prick test to an aeroallergen extended panel, total IgE and pulmonary function. Using questionnaires and clinical evaluation, participants were classified into the groups: chronic rhinitis alone (CRA) and chronic rhinitis + asthma, the latter subdivided into CR + mild asthma (CRMA) and CR + moderate to severe asthma (CRMSA). Aerollergen sensitization was defined by a positive prick test to one or more allergens associated with nasal symptoms and/or asthma. The association between CR and asthma was evaluated by multivariable logistic regression. The evidence of effect modification of pattern of sensitization in CR on the association with asthma severity and outcomes was examined by introducing interactions terms in the logistic regression models adjusting for confounders.

**Results:**

Frequency of sensitization to aeroallergens was higher in association with asthma in comparison to CRA (CRMA 70.4%; CRMSA 65.0%; CRA 47.0%; *p* = 0.000). Similarly, the presence of asthma was associated to aeroallergen multiple sensitization (51.5%) (OR = 2.10, 95% CI 1.27–3.50). Additionally, the sensitization to mites, cockroaches, animal epithelium, grasses, and molds, were higher in asthma (56.8%, 24.3%, 12%, 7.13% and 10.3%, respectively). Sensitization to *Alternaria alternata*, *Cladosporium herbarum* and dog epithelium was exclusive in asthma groups. A concomitant asthma diagnosis was directly associated with a positive allergen sensitization at least one allergen (62.7%, OR = 2.45, 95% CI 1.80–3.34) and polissensitization (51.5%, OR = 2.10, 95% CI 1.27–3.50).

**Conclusion:**

Asthma is associated with multiple allergen sensitization among patients with CR. Some unique profiles of aeroallergen sensitization were observed in patients with CR and asthma. Nevertheless, no difference was found in the sensitization in relation to asthma severity, which suggest atopy is not the main underlying mechanism for asthma severity among patients with CR.

**Level of evidence:**

Level 3.

## Highlights

• The chance of asthma in polysensitization individuals is increased.
• Allergic rhinitis is a relevant and independent risk factor for asthma development.
• Lower airway obstruction should be evaluated in patients with chronic rhinitis.
• Polysensitization and asthma are associated in a low-income setting in Brazil.

## Introduction

Chronic rhinitis (CR) is defined as an inflammatory condition that results in chronic dysfunction of the nasal mucosa,^1^ which can be found in over 80% of patients with asthma.^2^ Allergic rhinitis is the most common type of CR, some 10%–40% of patients with allergic rhinitis are diagnosed with asthma^3^ and allergic sensitization is a major risk factor for the development of asthma, both in adults and children.^4^

The association between atopy and asthma appears to be specific to sensitization to aeroallergens, and inhalable allergens have a greater clinical relevance, both in rhinitis and asthma. In Brazil, those originating from dust mites, cockroaches, fungi, animal epitelia and pollens are the most relevant.^5^

Recently, several studies have demonstrated the importance of allergic sensitization in childhood, as a high-risk predictor for the development of persistent asthma.^6^

Allergic rhinitis is a relevant and independent risk factor for asthma development, often preceding bronchial hyperreactivity^6^, ^7^ and asthma.^7^, ^8^ Although there are several studies demonstrating aeroallergen sensitization is associated with the risk of asthma,^8^, ^9^, ^10^ it is still unclear whether there is an association between sensitization and severity of asthma.

The aim of this study was to explore the differences in the pattern of allergen sensitization measured through skin prick test (SPT) in CR individuals without or with asthma, according to its severity. To the best of our knowledge, there has been no publication exploring this subject in the literature yet. The results of this study may clarify whether there is a specific sensitization profile in individuals with CR according to the presence of asthma and its severity.

## Methods

### Study population

This study is a sectional analysis nested in a cohort, performed according to standardised operating procedures,^10^, ^11^ that was carried out from 2013 to 2015. The classification of individuals with mild asthma was based on current concepts of severity by a doctor’s interview,^11^, ^12^ and classification to severe asthma was based on the presence of any of the following criteria: (I) report of daily symptoms, (II) frequent exacerbations or nocturnal symptoms, (III) limitation in their physical activities, (IV) reduced lung function (FEV1 or peak expiratory flow ≤60%) or FEV1 or peak expiratory flow variability > 30%. All individuals with moderate to severe asthma were being treated with a combination of medium or high doses of inhaled corticosteroids. Patients with severe asthma were followed for at least six months.

Recruitment criteria of Individuals without asthma and with mild asthma, and exclusion criteria of the cohort are well described in a recent publication of ours.^10^, ^11^

A total of 1066 subjects were included and classified into the groups chronic rhinitis (CR) alone (CRA, n = 212) and chronic rhinitis and concomitant asthma (n = 854). Individuals with CR and concomitant asthma were subdivided into CR + mild asthma (CRMA, n = 408) and CR + moderate to severe asthma (CRMSA, n = 446).

This study was approved by the Research Ethics Committee. All participants recruited and who agreed to participate in the study, accepted and signed the informed consent form.

### Chronic rhinitis diagnosis and atopy status

The diagnosis of CR was established through clinical evaluation by a specialist physician, with clinical history and physical examination compatible with the disease. The criteria used for the etiological definition and severity of allergic rhinitis in patients with asthma and individuals without asthma in the present study were those established by ARIA 2008,^12^, ^13^ in which the presence of symptoms such as sneezing, runny nose, nasal/ocular itching, as well as the intensity of symptoms and their duration are considered.

All patients included in the study were diagnosed with chronic rhinitis, and underwent the skin prick test for allergy with an extended panel, comprising the aeroallergens considered relevant in the Northeast of Brazil: *Aspergillus flavus*, *Aspergillus fumigatus*, *Aspergillus niger*, *Alternaria alternata*, *Cladosporium herbarum*, *Dermatophagoides farinae*, *Dermatophagoides pteronyssinus*, *Blomia tropicalis*, cat epithelium, dog epithelium, *Blatella germanica*, *Periplaneta americana*, *Paspalum notatum* and *Cynodon dactolon* (Immunotech Biopharm Ltd., Rio de Janeiro, Brazil). The SPT was performed as described in Bousquet et al. 2012 by pricking the skin of the forearm,^13^, ^14^ with a lancet through a drop of an allergen extract. The test reading occurred 15 min after the puncture, being considered positive when the papules had a mean diameter greater than 3 mm, after subtracting the diameter of the negative control. Those patients who presented positive SPT to at least one aeroallergen, and with a clinical history compatible with symptoms of CR associated to aeroallergens exposure were diagnosed to have allergic rhinitis (AR), and classified as a subgroup of CR.

### Statistical analysis

The statistical software of Statistical Product and Service Solutions (SPSS) version 26.0 (Armonk, NY: IBM Corp) and GraphPad Prism version 8.4 (GraphPad Software, SanDiego) were used to perform the statistical analysis. The distribution of variables was examined by the Kolmogorov–Smirnov test. Comparisons of continuous variables between 2 or more groups were performed using nonparametric methods, such as Mann–Whitney test and Kruskal–Wallis test, respectively. Furthermore, the Chi-square test was used to analyze categorical variables and the correlation between each variable and the SPT result. Clinical variables of interest were managed by logistical regression analysis. In addition, the results are expressed in median (first and third quartiles). All statistical tests were two-tailed, and the values of *p* < 0.05 was considered statistically significant.

## Results

### Study population characteristics

Table 1 shows the general characteristics of the sample. The median age was higher in the CRMSA group compared to CR alone (CRA) or with mild asthma (CRMA) (*p* < 0.001). The female sex was more frequent in all groups, mainly in CRA (90.1%). Family income was lower in the CRMSA group compared to the other groups and a greater median of the number of siblings was observed in this group compared to CRMA, but the same of CRA. In addition, the CRMSA group had a higher frequency of subjects with a body mass index (BMI) ≥ 30 kg/m^2^.

**Table 1:** Characteristics of the study population.

	CRA (n = 212)	CRMA (n = 408)	CRMSA (n = 446)	*p*
Age (years)^d^, ^e^	44 (33–42)	35 (26–46)	51 (42–61)	**<0.001**^a^**,**^b^**,**^c^
Female sex, n (%)^f^	191 (90.1)	318 (77.9)	364 (81.6)	**0.001**
BMI ≥ 30 kg m^2^, n (%)^f^	55 (16.6)	101 (30.4)	176 (53.0)	**0.001**
Total IgE (IU⁄mL)^d^, ^e^	165.6 (45.31–390.0)	207.9 (80.75–519.9)	331.6 (145.4–505.5)	**<0.001**^a^**,**^b^**,**^c^
Allergic Rhinitis^h^, n (%)^f^	87 (47.0)	264 (70.4)	262 (65.0)	**0.000**
Moderate-severe AR, n (%)^f^	78 (36.8)	227 (55.6)	191 (42.8)	**<0.001**
Persistent AR, n (%)^f^	46 (21.7)	167 (40.9)	133 (29.8)	**<0.001**

ACQ-6, Asthma Control Questionnaire, ACQ-6 ≤ 1.5 controlled asthma; AR, allergic rhinitis; BMI, body mass index; CRA, chronic rhinitis alone; CRMA, chronic rhinitis + mild asthma; CRMSA, chronic rhinitis + moderate to severe asthma; NA, not applicable; Pre-BD FEV1, pre-bronchodilator forced expiratory volume in one-second.

^g^Mann–Whitney test.

a CRMSA vs. CRA.

b CRMSA vs. CRMA.

c CRMA vs. CRA.

d Median (1st quartile‒3rd quartile).

e Kruskal–Wallis, Dunn’s post-test.

f Chi-square.

h Patients with valid SPT (CRA = 185; CRMA = 375; CRMSA = 403).

Regarding pulmonary function parameters, the pre-bronchodilator FEV1% predicted was lower in the CRMSA group compared to the other groups. We did not observe a significant difference in relation to the age of onset of the disease between the groups with asthma. The level of serum IgE was higher in both groups with asthma (CRMA and CRMSA) compared to the group without asthma (CRA), just as the level of IgE was higher in individuals with CRMSA than in subjects with CRMA. Asthma control was measured by the Asthma Control Questionnaire (ACQ-6). Patients with CRMA had better control compared to patients with CRMSA (*p* < 0.001). The highest frequence of AR was found in the CRMA group. As for the classification of AR, according to ARIA, CR with concomitant asthma had a higher frequency of moderate to severe and persistent rhinitis, compared to the group with only CR (*p* < 0.001) (Table 1).

### Aeroallergen sensitization profile in chronic rhinitis individuals with or without asthma

The frequency of positive SPT for different aeroallergens among patients with CR with or without asthma is shown in Table 2. The groups with CR and concomitant asthma (CRMA and CRMSA) had a higher frequency of positive tests for at least one aeroallergen compared to the CRA group. This increase was slightly accentuated for the CRMA group. Similarly, we observed that the positivity of most aeroallergens was higher in the CRMA and CRMSA groups, and the frequency of this positivity was also slightly higher in the CRMA group. However, of the 14 aeroallergens in the extended panel, 3 infrequently positive allergens did not show any significant difference between the groups: *A. flavus*, *A. niger* and *C. dactolon*. No positivity was observed to any of 3 aeroallergens (*A. alternata*, *C. herbarum* and dog epithelium) in the CRA group (Table 2).

**Table 2:** Characteristics of aeroallergen sensitization by skin prick test (SPT).

	CRA (n = 212)	CRMA (n = 400)	CRMSA (n = 441)	*p*
Invalid SPT, n (%)	27 (12.7)	25 (6.3)	38 (8.6)	**<0.001**
Positive to at least one, n (%)	87 (41)	264 (66)	263 (59.4)	**<0.001**
*Aspergillus flavus*, n (%)	5 (2.4)	16 (4.0)	11 (2.5)	0.362
*Aspergillus fumigatus*, n (%)	3 (1.4)	19 (4.8)	8 (1.8)	**0.014**
*Aspergillus niger*, n (%)	3 (1.4)	14 (3.5)	9 (2.0)	0.214
*Alternaria alternata*, n (%)	0 (0.0)	13 (3.5)	14 (3.2)	**0.027**
*Cladosporium herbarum*, n (%)	0 (0.0)	12 (3.0)	7 (1.6)	**0.027**
*Dermatophagoides farinae*, n (%)	55 (25.9)	192 (48.0)	175 (39.7)	**<0.001**
*Dermatophagoides pteronyssinus*, n (%)	49 (23.1)	189 (47.3)	162 (36.7)	**<0.001**
*Blomia tropicalis*, n (%)	37 (17.5)	182 (45.5)	152 (34.5)	**<0.001**
*Cat Epithelium*, n (%)	7 (3.3)	41 (10.3)	30 (6.8)	**0.006**
*Dog Epithelium*, n (%)	0 (0.0)	26 (6.5)	20 (4.5)	**<0.001**
*Blatella germânica*, n (%)	16(7.5)	86 (21.5)	70 (15.9)	**<0.001**
*Periplaneta americana*, n (%)	12 (5.7)	83 (20.8)	66 (15.0)	**<0.001**
*Paspalum notatum*, n (%)	3 (1.4)	28 (7.0)	20 (4.5)	**0.009**
*Cynodon dactylon*, n (%)	1 (0.5)	13 (3.3)	13 (2.9)	0.094

CRA, chronic rhinitis alone; CRMA, chronic rhinitis + mild asthma; CRMSA, chronic rhinitis + moderate to severe asthma; NA, not applicable; SPT, skin prick test. Chi-square test.

In order to better understand the profile of sensitization to aeroallergens between the groups, we divided them into 5 categories: mites, cockroaches, grasses, molds and epithelia of animals. We observed that the frequency of positivity to multiple aeroallergens, as well as to the established groups (mites, cockroaches, grasses, molds, and epithelia of animals) was higher among individuals with CR and asthma, mainly in the CRMA group, in relation to CRA group (Table 3).

**Table 3:** Characteristics of the sensitization.

	CRA (n = 212)	CRMA (n = 400)	CRMSA (n = 441)	*p*
Multiple sensitization^a^, n (%)	59 (27.8)	226 (56.5)	207 (46.9)	**<0.001**
Sensitization to mites, n (%)	74 (34.9)	246 (61.5)	232 (52.6)	**<0.001**
Sensitization to cockroaches, n (%)	21 (9.9)	107 (26.8)	97 (22.0)	**<0.001**
Sensitization to grasses, n (%)	4 (1.9)	31 (7.8)	29 (6.6)	**0.013**
Sensitization to molds, n (%)	9 (4.2)	40 (9.8)	37 (8.3)	**<0.001**
Sensitization to animal epithelia, n (%)	7 (3.3)	57 (14.2)	44 (10.0)	**<0.001**

CRA, chronic rhinitis alone; CRMA, chronic rhinitis + mild asthma; CRMSA, chronic rhinitis + moderate to severe asthma; SPT, skin prick test; Chi-square test.

a Multiple sensitization ≥2 aeroallergens.

In addition, we performed an association analysis to find out whether the presence of a profile of sensitization or sensitization to multiple aeroallergens could influence asthma severity, shown in Table 4, Table 5. We observed that, in general, aeroallergen sensitization increases the risk for the outcome chronic rhinitis and asthma. An individual is two times more likely to have asthma if he has sensitization to any aeroallergen, regardless the type and number (Table 4). However, this chance increases if sensitization is to cockroaches (OR = 2.91), and it is even higher if sensitization is to grasses or animal epithelium (OR = 3.99). These results were maintained when the adjusted analysis was performed for female gender, age, number of siblings (data not shown) and family income (data not shown) (Table 4).

**Table 4:** Association of the sensitization profile with the presence of asthma in individuals with chronic rhinitis.

	CRA (n = 212)	CRMA + CRMSA (n = 841)	Odds ratio (95% CI)	Odds ratio adjusted (95% CI)
Sensitization at least one allergen, n (%)	86 (40.6)	527 (62.7)	**2.45 (1.80–3.34)**	**2.39 (1.73–3.29)**
Multiple sensitization^a^, n (%)	59 (27.8)	433 (51.5)	**2.10 (1.27–3.50)**	**1.69 (1.41–2.01)**
Sensitization to mites, n (%)	74 (34.9)	478 (56.8)	**2.45 (1.79–3.36)**	**2.49 (1.79–3.47)**
Sensitization to cockroaches, n (%)	21 (9.9)	204 (24.3)	**2.91 (1.80–4.69)**	**2.72 (1.67–4.43)**
Sensitization to grasses, n (%)	4 (1.9)	60 (7.13)	**3.99 (1.43–11.11)**	**3.84 (1.36–10.81)**
Sensitization to molds, n (%)	9 (4.2)	87 (10.34)	**2.55 (1.26–5.17)**	**2.59 (1.27–5.27)**
Sensitization to animal epithelia, n (%)	7 (3.3)	101 (12.0)	**3.99 (1.83–8.73)**	**3.66 (1.67–8.11)**

CRA, chronic rhinitis alone; CRMA, chronic rhinitis + mild asthma; CRMSA, chronic rhinitis + moderate to severe asthma.

Adjusted to female gender, age, number of siblings and family outcome and calculated by binomial model.

a Multiple sensitization ≥2 aeroallergens.

**Table 5:** Association between the sensitization profile and asthma severity in individuals with chronic rhinitis.

	CRMA (n = 400)	CRMSA (n = 441)	Odds ratio (95% CI)	Odds ratio adjusted (95% CI)
Sensitization to ≥1 allergen, n (%)	264/400 (66.0)	263 (59.6)	0.76 (0.57–1.00)	1.27 (0.90–1.80)
Multiple sensitization^a^, n (%)	226/264 (85.6)	207 (78.7)	**0.62 (0.39–0.97)**	1.11 (0.93–1.34)
Sensitization to mites, n (%)	246 (61.5)	232 (52.6)	**0.69 (0.58–0.91)**	1.15 (0.82–1.63)
Sensitization to cockroaches, n (%)	107 (26.8)	97 (22.0)	0.77 (0.56–1.05)	1.07 (0.73–1.58)
Sensitization to grasses, n (%)	31 (7.8)	29 (6.6)	0.83 (0.49–1.41)	0.94 (0.48–1.80)
Sensitization to molds, n (%)	50 (12.3)	37 (8.3)	0.64 (0.41–1.01)	0.69 (0.40–1.19)
Sensitization to animal epithelia, n (%)	57 (14.2)	44 (10.0)	0.66 (0.43–1.01)	0.80 (0.48–1.34)

CRMA, chronic rhinitis + mild asthma; CRMSA, chronic rhinitis + moderate to severe asthma.

Adjusted to female gender, age, number of siblings and family outcome and calculated by binomial model.

a Multiple sensitization ≥2 aeroallergens.

We also assessed whether these sensitizations may be associated with asthma severity, and we noted that, individuals who have sensitized to more than 2 aeroallergens or sensitized to mites were less likely to have the severe form disease. Thus, apparently, aeroallergen sensitization among asthmatic subjects with CR was inversely associated with severe asthma, but the association did not have remain in the adjusted analysis (Table 5).

## Discussion

In this study we assessed the profile of sensitization to aeroallergens through SPT in individuals with chronic rhinitis, with or without asthma, as well as the main clinical characteristics associated with asthma severity. Our data showed a lower frequency aeroallergen sensitization in individuals with chronic rhinitis, with a positive association for polysensitization in the presence of asthma, even after adjusting for potential confounders. Regarding the severity of asthma, no significant difference was found considering the profile of aeroallergens.

In recent years, there has been increased interest in the study of the association between asthma and rhinitis, since both illnesses present similar pathophysiological mechanisms, involving nasal and bronchial dysfunction,^9^, ^10^ but to consider them as a single disease may be an oversimplification.

We observed that the majority of patients with chronic rhinitis and concomitant asthma are sensitized to aeroallergens (Table 4), similar to that previously described by other studies, in which more than 70% of patients are sensitized to more than one aeroallergen, both in the adult and pediatric population.^14^, ^15^, ^16^, ^17^, ^18^ We found a more frequent polysensitization in patients with CR and concomitant asthma, as well as Siroux et al., who assessed the profile of sensitization by serum specific IgE, and found high frequency of polysensitization in adults and adolescents with rhinitis and asthma.^18^, ^19^ These results are consistent with other studies that evaluated the profile of allergic sensitization, including patients with severe asthma.^19^, ^20^, ^21^ Nevertheless, no association was found between polysensitization and asthma severity in our sample. Similarly, Ponte et al., showed that positive SPT was not a predictor of asthma control neither of worse prognosis in adults with asthma.^21^, ^22^

In our study we observed that the presence of more persistent and more severe symptoms of allergic rhinitis occurred among individuals with chronic rhinitis associated with asthma compared to those with chronic rhinitis alone (Table 1). This corroborates with Bousquet et al., that showed in the Allergic Rhinitis and its Impact on Asthma (ARIA) update that the persistence of allergic disease is most often associated with various comorbidities, in addition to aeroallergen polysensitization.^12^, ^13^ It is worth to note that polysensitization, and not only aeroallergen sensitization, was associated with the presence of asthma in our study. Similarly, Burte et al., showed that individuals with allergic rhinitis having a high rate of polysensitization had a higher chance of presenting other manifestation of atopy, such as allergic conjunctivitis and atopic asthma.^17^, ^18^

To explain the interaction between upper and lower airways, particularly the relationship between allergic rhinitis and asthma, several mechanisms have been proposed.^22^, ^23^ Among these mechanisms, a crucial role of upper airway disease inducing and maintaining lower airway disease was demonstrated by the presence of thickening of the basement membrane of the epithelium, a typical marker of remodeling of the lower airway, not only in patients with asthma, but also in atopic patients without asthma and patients with allergic rhinitis.^23^, ^24^

The impact of the presence and severity upper airway disease on asthma severity and poor control is evidente.^22^, ^25^, ^26^ Regarding asthma severity and/or control and allergic rhinitis, a trial conducted by Bousquet et al., found that the presence of self-reported allergic rhinitis in patients with asthma resulted in a higher rate of asthma attacks in comparison to patients with asthma without concomitant allergic rhinitis.^27^ In addition, patients with concomitant allergic rhinitis and asthma have more asthma attacks and emergency visits.^22^, ^28^ Furthermore, nasosinusal symptoms severity is also closely associated with asthma control status (e.g., ACT score, FEV1 and acute exacerbations) among patients with asthma and persistent nasal symptoms.^22^, ^29^, ^30^

Regarding phenotypic characteristics of asthma, it is known that late onset asthma is less associated with atopy, has a worse prognosis, besides poor responsiveness to treatment, as compared to individuals with childhood-initiated asthma.^31^ The age of onset asthma symptoms is considered early when it started less than 12 years old,^32^ and our population had a median less than this age (7 years old for CRMA and 10 for CRMSA; Table 1). This type of patient tends to have a strong association between allergen exposure and asthma symptoms,^33^ and between upper and lower airway symptoms.^34^, ^35^ Both asthma and allergic diseases such as rhinitis, are commonly developed in childhood, and studies of birth cohorts have been shown that allergic sensitization in childhood represents a risk factor for the development of both diseases,^36^ and children delivered by cesarean section with parental history of asthma had an increased risk of chronic rhinitis and allergic rhinitis.^37^ The polysensitization phenotype associated with early onset asthma starts in the first years of life, and seems to persist throughout life.^38^

Another way to assess the presence of allergy is by dosage of serum levels of total IgE, values above 160 IU/mL are considered suggestive of allergy.^11^, ^12^ We noticed that these levels were significantly higher in the groups with concomitant rhinitis and asthma (Table 1), consistent with what was demonstrated in the study of Burte et al.^24^, ^39^ The authors observed a higher level of total serum IgE in the allergic rhinitis and asthma groups, compared to groups without asthma or rhinitis, or with non-allergic rhinitis only,^24^, ^39^ indicating a greater tendency to allergic phenotype in these individuals. This phenotype of allergic rhinitis and asthma is associated with eosinophilia, high levels of serum IgE and cytokine of the Th2 profile.^40^

Additionally, we found that the prevalence and severity of allergic rhinitis was significantly higher in the groups with concomitant rhinitis and asthma (Table 1). Orlandi et al. have also suggested that asthma is more common in patients with persistent moderate-severe rhinitis, compared to those with mild rhinitis.^3^ It had already been previously reported by Ponte et al., that found in a population with severe asthma, moderate to severe rhinitis is a stronger predictor for greater severity of asthma.^21^, ^22^

In relation to asthma control, our findings re-reinforce the data from study of Ponte et al., in which it showed concomitant rhinitis in asthma, impacts both on asthma control, when it's moderate to severe rhinitis, and increasing the risk to emergency room visits for acute asthma, regardless of the severity of the rhinitis.^22^ Additionally, our results are similar to a recent study conducted with Chinese adults with asthma, which reported a higher prevalence of poor asthma control among those with nasal diseases.^41^

In the present study we also evaluated the profile of sensitization to mites, cockroaches, grasses, mold, and epithelia of animals. As for the profile of sensitization to mites (*D. pteronyssinus*, *D. farinae* and *B. tropicalis*), we found a high frequency of sensitization in all patients with chronic rhinitis with concomitant asthma, mainly in the CRMA group (Table 3). Several studies have pointed out that the sensitization to mites is clearly identified as a risk factor for asthma, as well as for its severity. Virot et al. evaluated the correlation between symptoms of patients with allergic rhinitis and/or asthma and sensitization to household aeroallergens and found that sensitization to mites was associated with severe asthma.^20^, ^21^ But in our study we did not observe a similar finding (Table 4, Table 5). We found that presence of polysensitization or sensitization to mites, as well as sensitization to any of the allergens evaluated were associated with increased chances of concomitant asthma. Kovac et al., reported high levels of serum specific IgE for D. pteronyssinus in children with severe asthma.^42^ Likewise, Sylvestre et al. observed a positive correlation between dust mite sensitization and asthma severity.^43^

Regarding the sensitization profile of other household allergens, we investigated two species of cockroaches (*B. germanica* and *P. americana*), sensitization to dog and cat epithelium. Sensitization to cockroach allergens has been identified as one of the strongest risk factors for the development of asthma in low-income urban populations, such as the population of our study.^25^, ^44^ Sensitization to dog epithelium was found only in individuals with asthma in the present study. This result is consistent with the report by Uriarte and Sastre, which found sensitization to dogs in the group with allergic rhinitis and asthma and that sensitization to two or more dog allergens was associated with severe asthma.^26^, ^45^ There are still few relevant clinical studies with molecular assessment of sensitization to aeroallergens in animals such as dogs and cats, and studies that evaluated this type of allergy derived from animals in children, showed a significant association between the severity of rhinitis and asthma.^27^, ^28^, ^46^, ^47^

As for sensitization to grasses, our data showed an increase in risk for the development of asthma in patients with chronic rhinitis of approximately four times. Grasses are recognized as one of the most common seasonal aeroallergens found in the environment, and in recent studies, such as Ahmed et al.,^29^, ^48^ grasses sensitization among individuals with asthma and rhinitis was 39.2%, much higher than that found in our study, that was approximately 7%.^29^, ^48^ This can be explained by the fact that the study by Ahmed et al. was carried out in a subtropical region (Canada), in which there are well-defined climatic seasons, with a pollination period, unlike what occurs in Brazil, especially in the northeast region, where our study was performed, in which we have basically two annual seasons, with high temperatures in the most part of the time, and grass pollen allergy has not been reported as clinically relevant problem.

Molds also represents an important group of indoor aeroallergens associated with respiratory allergy.^30^, ^49^ In our study the sensitization to molds was greater in individuals with rhinitis and asthma, representing an increase of approximately two times in the chance of the asthma outcome (Table 4). It has already been described that fungal allergy has a direct association with the asthma severity, and that long-term fungal infections are associated with poor asthma control, in addition to complications such as bronchiectasis and chronic allergic bronchopulmonary aspergillosis (ABPA).^50^ None of the four groups of our present study presented any case of ABPA, however.

In the present study we assessed the most frequent molds found in respiratory allergic conditions — *Cladosporium* sp., *Aspergillus* sp., *Alternaria* sp. and *Penicillium notatum*. Our fungal sensitization rate (Table 3) was similar to those described in the literature, between 7% to 20%, but lower than that described in the group associated with severe asthma, that was between 35% to 75%.^31^, ^32^, ^50^, ^51^ This percentage discrepancy may be due to the tropical climatic conditions of Northeastern Brazil, which is a type of climate with a higher prevalence of sensitization to mites,^33^, ^52^ as well as for the different methodologies used in each study (immediate hypersensitivity skin test in this study versus specific serum IgE measurements for molds in the study by O’Driscoll et al.).^32^, ^51^ Interestingly, it was shown that sensitization to *C. herbarum* and *A. alternata* were found only among subjects with asthma in our sample, which agrees with previous studies in the literature.^34^, ^35^, ^36^, ^53^, ^54^, ^55^

Finally, we demonstrated an increased chance of presenting asthma among individuals with chronic rhinitis who had polysensitization and some specific type of aeroallergen sensitization, corroborating the understanding that allergic rhinitis is a relevant and independent risk factor for the development of asthma,^37^, ^56^ reinforcing that lower airway obstruction should be evaluated in patients with chronic rhinitis.^7^, ^8^

Our study is limited by being observational, cross-sectional, which does not allow inferring causality, in addition to being subject to bias. However, a sample of the patients in this first study underwent an otorhinolaryngological evaluation, to differentiate it from other possible upper airway comorbidities, mainly chronic rhinosinusitis with or without nasal polyposis, which symptoms may overlap with those of chronic rhinitis, for a better correlation of the impact of upper airway pathologies in asthma.

## Conclusion

The relevance of this study is highlighted, consisting of one of the largest samples of patients with severe asthma in the country, in addition to the unprecedented investigation of the profile of sensitization to aeroallergens through immediate skin hypersensitivity test in adult patients with asthma and individuals without asthma in northeast region of Brazil. In this way, this study can inform clinical care personalized decisions.

In summary, our findings reiterate that aeroallergen sensitization is relevant to asthma. Among patients with chronic rhinitis, sensitization to aeroallergens in general, to aeroalergens of different categories and polysensitization is associated with a higher odd of having asthma in a low-income tropical setting in Brazil.

## Funding

This work was supported by Capes and CNPQ (PRONEX – grant number PNX0018/2009 of 10.13039/501100006181FAPESB). This study was approved by the Research Ethics Committee of Maternidade Climério de Oliveira, Federal University of Bahia (License Number: CONEP nº 15782, CEP 095⁄2009, 095⁄2012, 032⁄2014). All participants recruited and who agreed to participate in the study, accepted and signed the informed consent form.

## Conflicts of interest

The authors declare no conflicts of interest.
